# Beyond Shedding: Conserved Retention of ZAN After the Acrosome Reaction Across Rabbit, Boar, and Bull Sperm

**DOI:** 10.3390/ijms27188062

**Published:** 2026-09-10

**Authors:** Miranda Hernández-Falcó, Lucía Díaz-Fuster, Núria Ferrer-Cortés, Laura Robles-Gómez, Paula Sáez-Espinosa, María José Gómez-Torres

**Affiliations:** 1 Departamento de Biotecnología, Facultad de Ciencias, Universidad de Alicante, 03690 Alicante, Spain; miranda.hernandez@ua.es (M.H.-F.); lucia.diaz@ua.es (L.D.-F.); nuria.ferrer@ua.es (N.F.-C.); laura.robles@ua.es (L.R.-G.); paula.saez@ua.es (P.S.-E.); 2 Cátedra Human Fertility, Facultad de Ciencias, Universidad de Alicante, 03690 Alicante, Spain

**Keywords:** acrosomal reaction, capacitation, fertilization, fusion, gamete interaction, mammals, spermatozoa, ZAN (zonadhesin)

## Abstract

Zonadhesin (ZAN) is a sperm protein currently known to mediate binding to the oocyte zona pellucida. Although its localization has been described in ejaculated and capacitated spermatozoa from mice, pigs, and rabbits, its distribution following the acrosome reaction remains poorly understood. Here, we characterized the localization of ZAN in rabbit, boar, and bull spermatozoa under in vitro conditions designed to mimic early fertilization events. Ejaculated spermatozoa, spermatozoa selected by swim-up in capacitation medium and spermatozoa subjected to acrosome reaction induction were analyzed. Immunofluorescence was performed using an antibody targeting a domain of ZAN distinct from the previously studied domain, together with PNA-FITC to assess acrosomal status; protein abundance was assessed by quantitative LC-MS/MS. ZAN was detected mostly in the anterior region of intact spermatozoa, consistent with previous reports, but the distinct epitope also revealed post-acrosomal localization. Species-specific patterns were observed in intact spermatozoa, whereas following the acrosome reaction, ZAN was consistently detected in the post-acrosomal region. These findings identify a previously unrecognized post-acrosomal distribution of ZAN and suggest that this may contribute not only to sperm–ZP binding but also to later stages of fertilization, including sperm–oolemma interaction and membrane fusion.

## 1. Introduction

During mammalian fertilization, zonadhesin (ZAN) plays an important role in sperm binding to the zona pellucida (ZP) of the oocyte. ZAN was originally isolated and characterized from porcine sperm as a large protein of approximately 300–350 kDa. This mosaic protein contains MAM-type domains (meprin/A5 antigen/tyrosine phosphatase μ receptor), a mucin-like domain, a von Willebrand factor-homologous D domain, and an epidermal growth factor (EGF) domain [1]. ZAN has been detected in several mammalian species [2], including pigs [1,3,4], mice [5,6], humans [7], rabbits [8], and hamsters [9]. Despite some interspecific variation in the number of D domains, the MAM-type domain is highly conserved, including four conserved cysteine residues and conserved hydrophobic/aromatic residues, which are crucial for maintaining its structure and potential role in adhesion [5].

Each of these domains may contribute to cell adhesion [6,8], either through interactions between proteins or between proteins and carbohydrates. According to Gao and Garbers (1998) [5], the mucin-like domain of ZAN may prevent nonspecific interactions between spermatozoa and cells of the oviductal isthmus, whereas the EGF domain may facilitate binding to the ZP. Consistent with an association between ZAN and the ZP, Lea et al. (2001) [8] detected ZAN in acrosomal remnants attached to the ZP following the acrosome reaction. Although sperm from Zan-deleted mice were less likely to recognize the ZP, they were not infertile, suggesting functional redundancy or compensatory mechanisms [6].

Electron microscopy studies in pigs [4] and hamsters [9], using antibodies that recognize the functional form of ZAN, localized the protein predominantly to the outer acrosomal membrane during spermiogenesis. In hamster round spermatids, however, ZAN was also detected in the inner acrosomal membrane before becoming largely restricted to the outer acrosomal membrane in late spermatids. In addition, weak labeling of the equatorial segment has been reported in intact rodent spermatozoa, suggesting species-specific differences in ZAN localization. Nevertheless, ZAN has been reported to become exposed during capacitation and has been proposed as a marker of this process [10]. To explain these findings, a kiss-and-run mechanism has been proposed, involving transient fusion pores between the plasma and acrosomal membranes that allow surface exposure of ZAN without complete acrosomal exocytosis.

The exposure of ZAN during capacitation raises the question of whether its localization undergoes further changes during subsequent stages of fertilization. In this context, other sperm proteins involved in ZP binding, such as CRISP1, have been shown to migrate to the equatorial region during the acrosome reaction, where they participate in sperm–oocyte fusion [11].

Despite advances in the study of ZAN, limited information is available on its interaction with other proteins during gamete recognition. Moreover, it remains unclear how its exposure during the acrosome reaction could facilitate sperm adhesion and penetration of the ZP. A detailed understanding of the molecular mechanisms that regulate the reorganization of membrane proteins on the surface of spermatozoa remains a challenge [12]. Sperm capacitation and fertilization are highly complex processes involving multiple interconnected proteins and biochemical events [13], making it challenging to isolate the specific role of ZAN [10]. Furthermore, studies of ZAN are complicated by interspecies variability and methodological limitations [12].

To overcome these knowledge gaps, additional studies using complementary techniques, such as immunocytochemistry and immunofluorescence, are needed to more precisely determine the distribution of ZAN in spermatozoa. A comparative approach across species may further help distinguish conserved from species-specific patterns in the localization of proteins involved in sperm–oocyte interactions, providing broader insight into the molecular events underlying mammalian fertilization [14]. In this context, rabbit, pig, and cattle provide a suitable comparative framework because they are phylogenetically distinct mammalian species. They are also of considerable zootechnical importance, with reproductive efficiency representing a key factor in the optimization of breeding programs and the sustainability and economic efficiency of their production systems [15,16,17]. Accordingly, the present study investigated ZAN using a multispecies comparative framework under in vitro conditions designed to mimic early fertilization events, specifically capacitation and the acrosome reaction. Furthermore, complementary analytical approaches were integrated to provide a comprehensive characterization, using fluorescence microscopy to assess ZAN distribution across distinct sperm head domains and proteomic analysis for quantitative comparison.

Overall, our findings show that ZAN remains detectable in mammalian sperm following the acrosome reaction, with consistent post-acrosomal localization across the three species examined. This previously underappreciated distribution provides a basis for further investigation of ZAN’s potential role during the final stages of fertilization, particularly during sperm–oolemma interactions.

## 2. Results

### 2.1. Evaluation of Acrosomal Status

**Figure 1:**
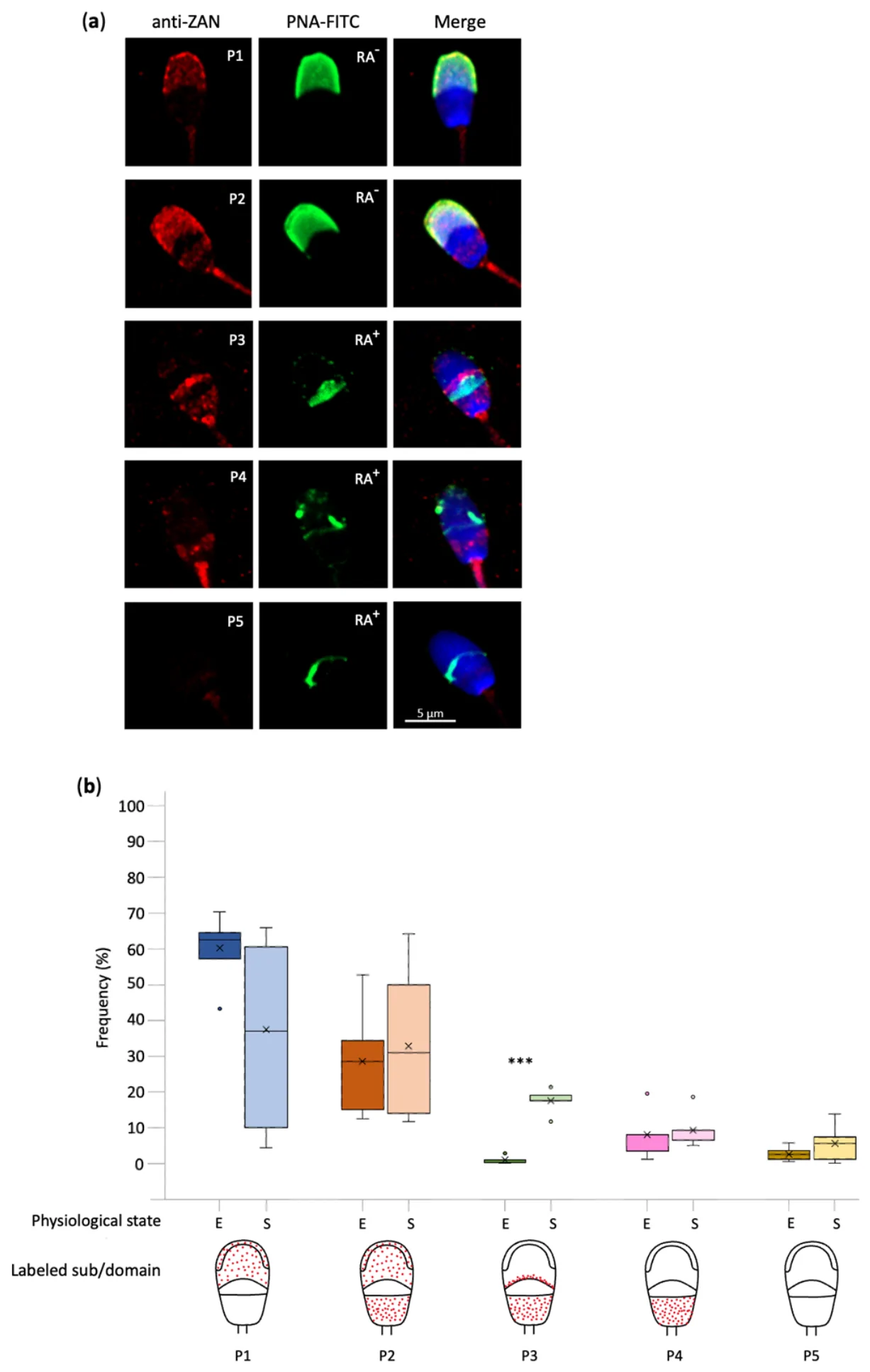
Analysis and distribution of ZAN staining patterns in rabbit sperm. (**a**) Representative images of ZAN and acrosome status co-staining. (**b**) Percentages of ZAN staining patterns in ejaculated spermatozoa (E) and spermatozoa selected by swim-up in capacitation medium (S), including both acrosome-intact and spontaneously acrosome-reacted spermatozoa. P1, speckled staining in the apical ridge and pre-equatorial subdomain; P2, strong staining of the apical ridge and pre-equatorial subdomain, together with speckled staining in the post-acrosomal domain; P3, staining at the equatorial bulges and in the post-acrosomal domain; P4, staining only in the post-acrosomal domain; P5, without labeling. Red dots indicate the localization of ZAN. Scale bar: 5 μm, common to all images. Significant differences at *p* < 0.001 (***) level, according to the Mann–Whitney U test.

**Figure 2:**
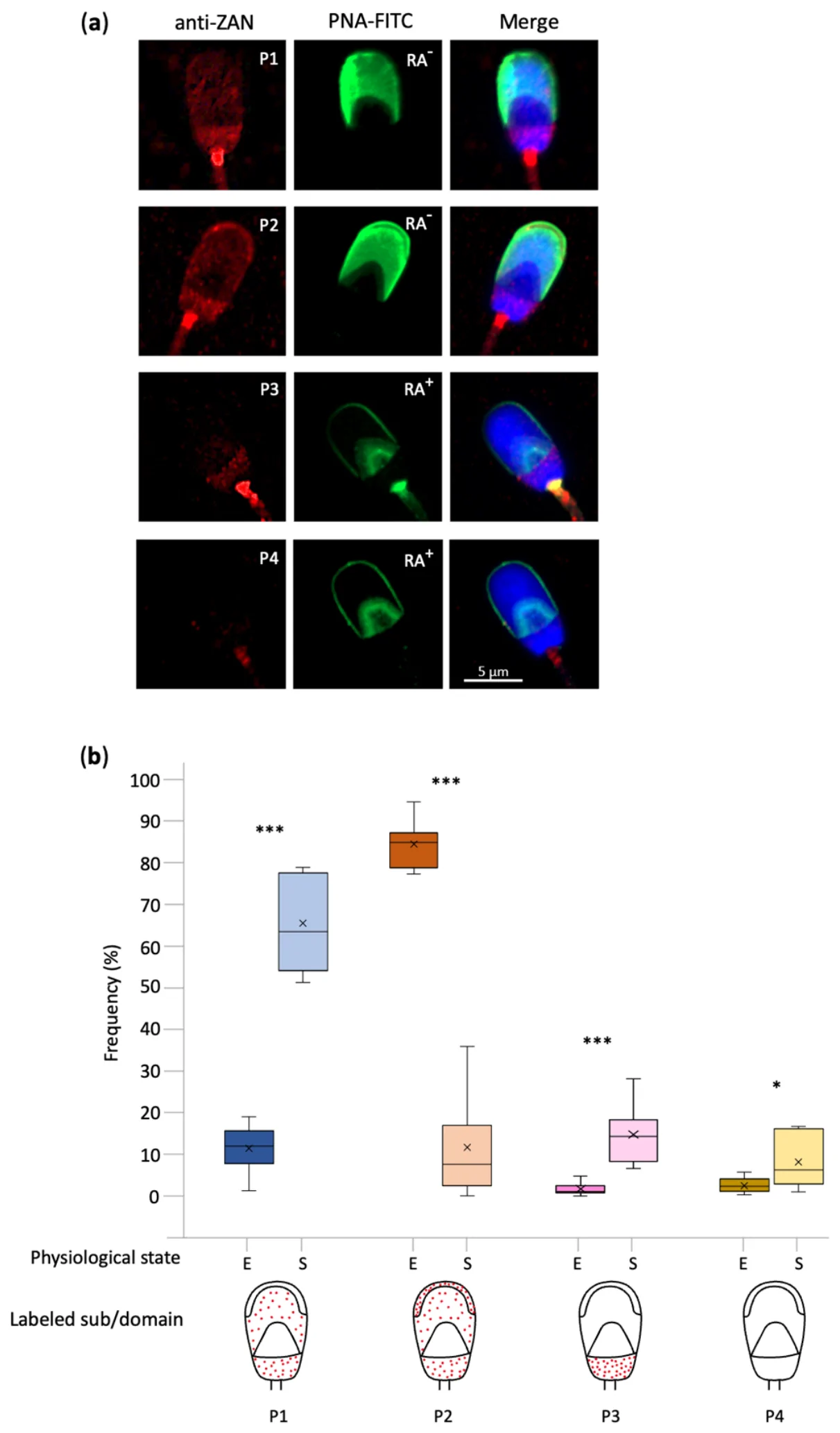
Analysis and distribution of ZAN staining patterns in boar sperm. (**a**) Representative images of ZAN and acrosome status co-staining. (**b**) Percentages of ZAN staining patterns in ejaculated spermatozoa (E) and spermatozoa selected by swim-up in capacitation medium (S), including both acrosome-intact and spontaneously acrosome-reacted spermatozoa. P1, speckled staining in the pre-equatorial subdomain and post-acrosomal domain; P2, strong apical ridge staining with speckled staining in the pre-equatorial subdomain and post-acrosomal domain; P3, speckled staining in the post-acrosomal domain; P4, without labeling. Red dots indicate the localization of ZAN. Scale bar: 5 μm, common to all images. Statistical significance was assessed using Student’s t-tests or Welch’s t-test, as determined by homogeneity of variances (Levene’s test). Significant differences at *p* < 0.05 (*) and *p* < 0.001 (***).

**Figure 3:**
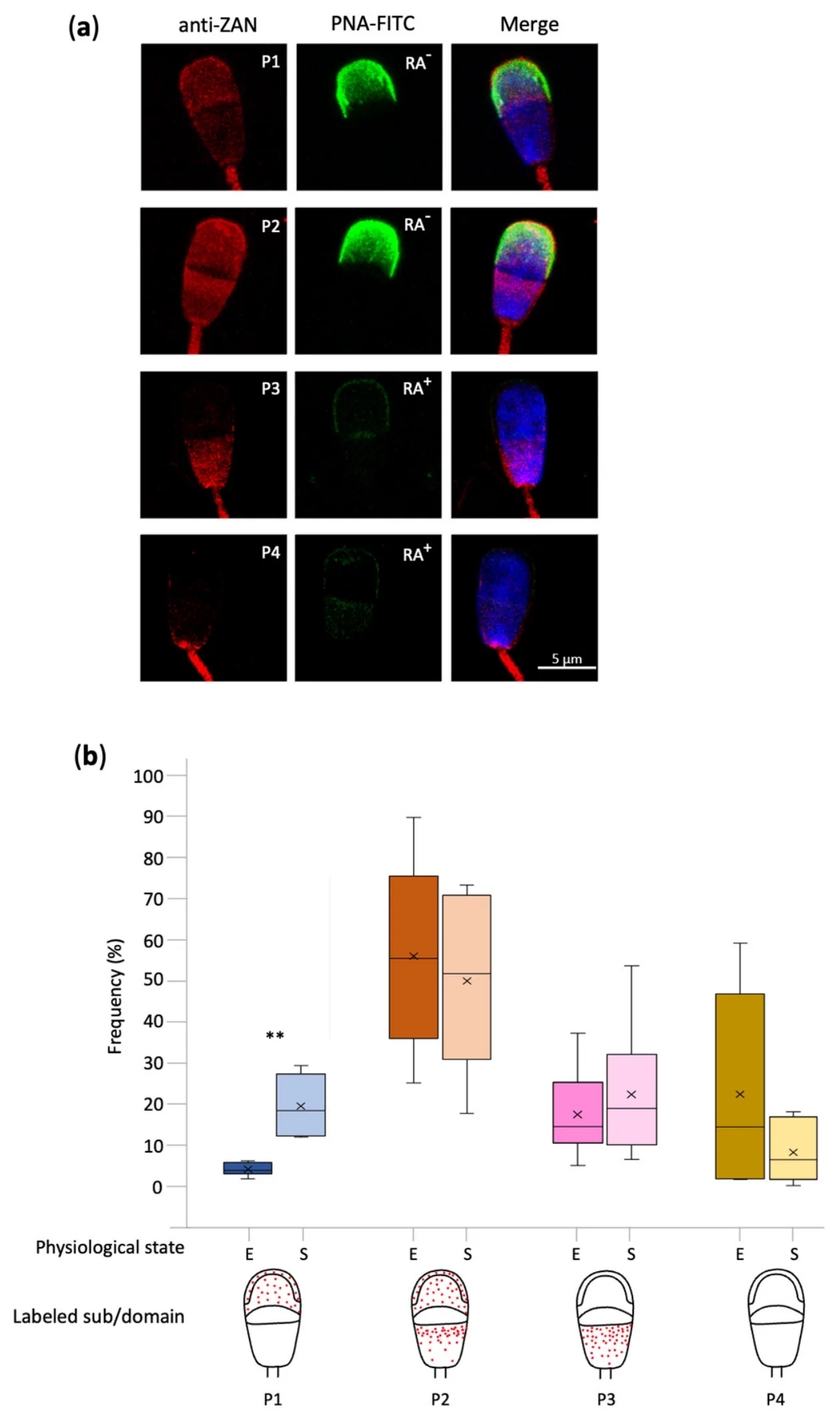
Analysis and distribution of ZAN staining patterns in bull sperm. (**a**) Representative images of ZAN and acrosome status co-staining. (**b**) Percentages of ZAN staining patterns in ejaculated spermatozoa (E) and spermatozoa selected by swim-up in capacitation medium (S), including both acrosome-intact and spontaneously acrosome-reacted spermatozoa. P1, speckled staining in the apical ridge and pre-equatorial subdomain; P2, strong apical ridge and pre-equatorial subdomain staining with speckled staining in the post-acrosomal domain; P3, staining only in the post-acrosomal domain; P4, without labeling. Red dots indicate the localization of ZAN. Scale bar: 5 μm, common to all images. Statistical significance was assessed using Student’s t-tests or Welch’s t-test, as determined by homogeneity of variances (Levene’s test). Significant differences at *p* < 0.01 (**).

Cells showing green fluorescence in the acrosome were classified as non-reacted, whereas cells showing labeling restricted to the equatorial region or no acrosomal labeling were classified as reacted (see Figure 1a, Figure 2a and Figure 3a). No significant differences in spontaneous acrosome reaction rates were observed between ejaculated spermatozoa and spermatozoa subjected to swim-up selection in capacitation medium in rabbit and bull spermatozoa (rabbit E: 10.23% vs. S: 28.95%; bull E: 1.83% vs. S: 3.17%), whereas a significant difference was observed in boar spermatozoa (E: 4.00% vs. S: 22.83% *p* < 0.01). Induction of the acrosome reaction significantly increased the proportion of acrosome-reacted spermatozoa compared with ejaculated samples in rabbit, boar, and bull (E vs. AR; *p* < 0.01). Moreover, a significant increase was observed between S and AR in rabbit (28.95% vs. 85.85%; *p* < 0.05) and boar (22.83% vs. 73.76%; *p* < 0.001), whereas no significant difference was observed in bull spermatozoa (3.17% vs. 16.50%; *p* > 0.05).

### 2.2. Permeabilization Reveals ZAN in Post-Acrosomal Domain of Rabbit Intact Spermatozoa

The analysis of ZAN distribution in rabbit sperm revealed five different staining patterns (Figure 1a): pattern 1 was characterized by speckled staining in the apical ridge and pre-equatorial subdomain; pattern 2 exhibited strong staining of the apical ridge and pre-equatorial subdomain, together with speckled staining in the post-acrosomal domain; pattern 3 showed staining at the equatorial bulges and in the post-acrosomal domain; pattern 4 displayed staining only in the post-acrosomal domain; and pattern 5 showed no labeling.

Following swim-up selection in capacitation medium, the proportion of spermatozoa showing ZAN localization at both the equatorial bulges and the post-acrosomal domain increased significantly from 0.99% to 17.59% (pattern 3; Mann–Whitney U test; *p* = 0.001). Conversely, no significant differences were observed in the remaining patterns (Mann–Whitney U test, *p* > 0.05) when comparing ejaculated sperm with those selected in capacitation medium: pattern 1 (62.58% vs. 37.03%), pattern 2 (28.53% vs. 30.97%), pattern 4 (8.02% vs. 9.18%), and pattern 5 (2.53% vs. 5.50%; see Figure 1b for details).

### 2.3. Dynamic Remodeling of ZAN Patterns During Swim-Up Selection in Capacitation Medium in Porcine Sperm

Given the characteristic organization of membrane domains in porcine sperm cells, ZAN exhibited the same four localization patterns previously described by our group for the IZUMO1 protein [18], though their relative abundances were different. It should be noted that the patterns were as follows (Figure 2a): pattern 1 was characterized by speckled staining in the pre-equatorial subdomain and post-acrosomal domain, pattern 2 displayed strong apical ridge staining with speckled staining in the pre-equatorial subdomain and post-acrosomal domain, pattern 3 exhibited speckled staining in the post-acrosomal domain, and pattern 4 showed no labeling.

Sperm swim-up selection in capacitation medium induced significant changes in the relative distribution among the four localization patterns (*p* < 0.05 for each pattern; Figure 2b). For the variables meeting the assumption of equal variances, independent samples t-tests showed that spermatozoa displaying strong apical ridge staining with speckled staining in the pre-equatorial subdomain and post-acrosomal domain were significantly higher in ejaculated spermatozoa than in spermatozoa selected in capacitation medium (pattern 2: from 84.54% to 11.63%; Student’s t-tests; *p* < 0.001); meanwhile those spermatozoa exhibiting speckled staining only in the post-acrosomal domain were higher after selection in capacitation medium (pattern 3: from 1.64% to 14.70%; Student’s t-tests; *p* < 0.001).

For variables with unequal variances, Welch’s t-tests revealed that spermatozoa with labeling in the pre-equatorial subdomain and post-acrosomal domain were significantly higher after selection in capacitation medium (pattern 1: from 11.46% to 65.54%; Welch’s t-test; *p* < 0.001), as was the proportion of spermatozoa without labeling (pattern 4: from 2.36% to 8.13%; Welch’s t-test; *p* = 0.038).

### 2.4. Swim-Up Selection in Capacitation Medium Enhances ZAN Protein Localization in the Acrosomal Domain of Bull Sperm

Upon analysis of the ZAN distribution in bull sperm, the following four staining patterns were observed (Figure 3a): pattern 1 was characterized by speckled staining in the apical ridge and pre-equatorial subdomain; pattern 2 exhibited strong apical ridge and pre-equatorial subdomain staining with speckled staining in the post-acrosomal domain; pattern 3 displayed staining only in the post-acrosomal domain; and pattern 4 showed no labeling.

Comparison of ejaculated and swim-up-selected bull sperm in capacitation medium (Figure 3b) revealed a significant difference only in the distribution of pattern 1 characterized by speckled staining in the apical ridge and pre-equatorial subdomain with an increase from 4.21% in E to 19.55% in S (Welch’s t-test; *p* < 0.001). No significant differences were observed for apical ridge and pre-equatorial subdomain staining with speckled staining in the post-acrosomal domain (pattern 2; E: 55.91% vs. S: 49.81%), or for pattern 3, characterized by staining only in the post-acrosomal domain (E: 17.44% vs. S: 22.29%), as assessed by Student’s t-tests (*p* > 0.05). Similarly, the proportion of spermatozoa showing no labeling (pattern 4) did not differ significantly between groups (E: 22.44% vs. S: 8.35%; Welch’s t-test; *p* > 0.05).

### 2.5. Conservation of ZAN Following the Acrosome Reaction in Mammalian Sperm

**Figure 4:**
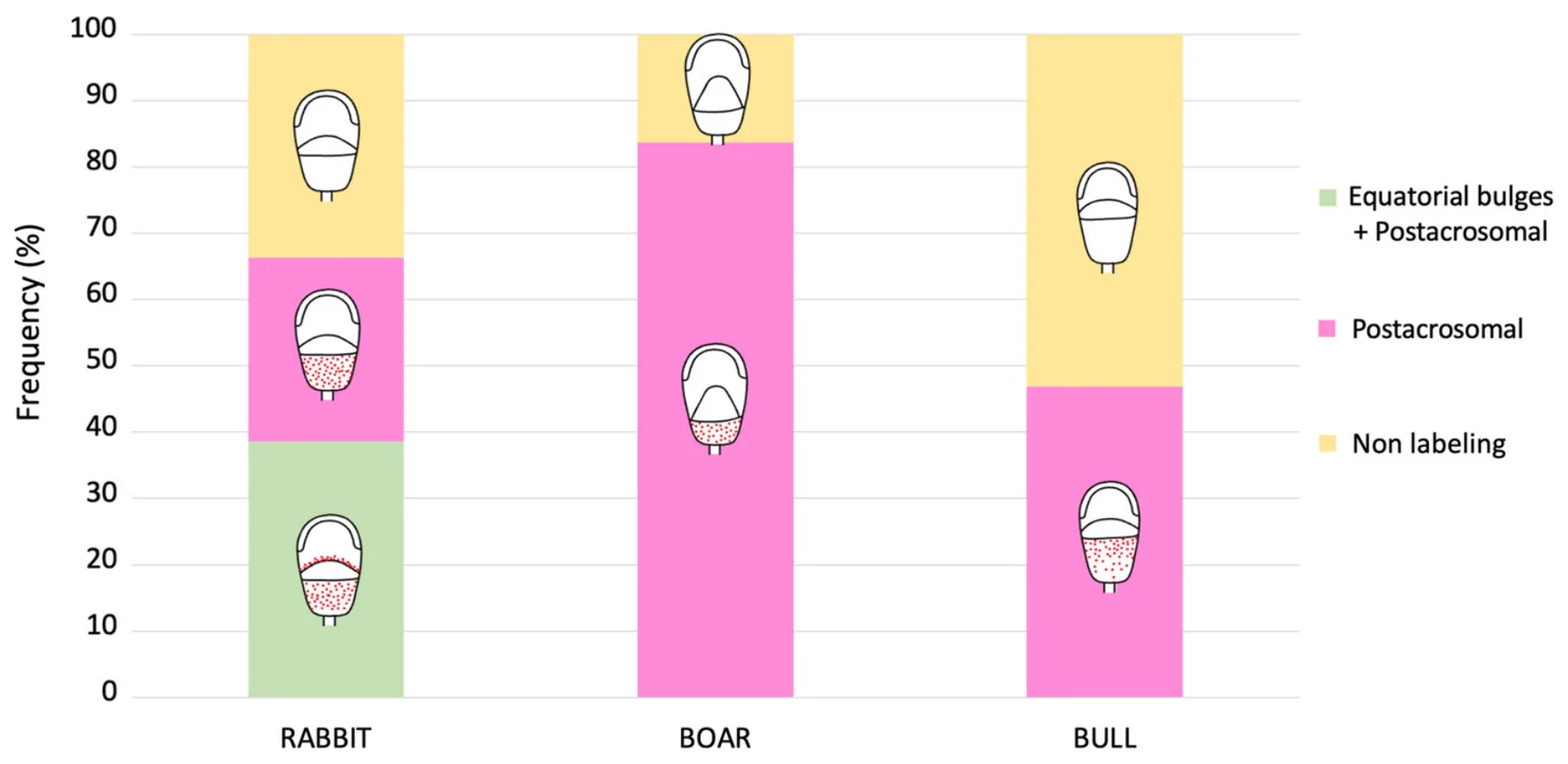
ZAN distribution following the acrosome reaction in rabbit, boar, and bull spermatozoa. Red dots indicate the localization of ZAN.

To determine whether ZAN remained detectable after induction of the acrosome reaction, only spermatozoa confirmed as acrosome-reacted by PNA-FITC staining were included in the analysis. ZAN was not detectable in a subset of acrosome-reacted spermatozoa in all three species analyzed (Figure 4). Specifically, the absence of ZAN was detected in 33.62% of rabbit spermatozoa, 16.30% of boar spermatozoa, and 53.14% of bull spermatozoa. Conversely, spermatozoa that retained ZAN after the acrosome reaction exhibited localization predominantly within the post-acrosomal domain, accounting for 66.38% in rabbit (pattern 3 + pattern 4), 83.70% in boar, and 46.86% in bull spermatozoa. Moreover, 38.7% of rabbit spermatozoa also showed labeling of the equatorial bulges (pattern 3). Notably, patterns 1 and 2 were not detected among the acrosome-reacted spermatozoa analyzed in any of the three species.

### 2.6. Quantitative Analysis of ZAN Protein

**Figure 5:**
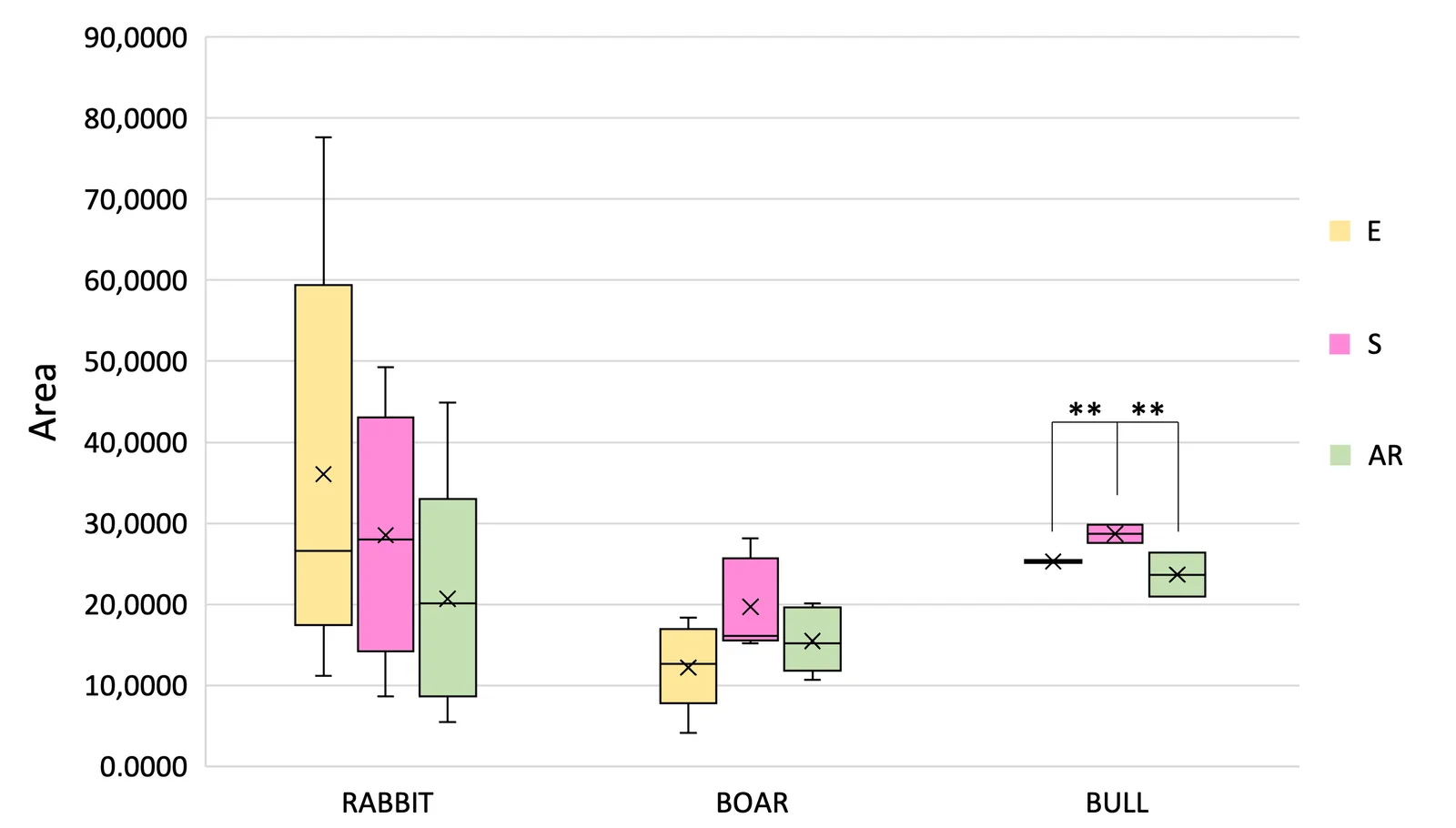
Relative abundance of ZAN across ejaculated sperm (E), selected via swim-up in capacitation medium (S) and acrosome reaction-induced (AR) states in rabbit, boar, and bull sperm. Data represent median peak area. Statistical significance was assessed using one-way ANOVA (rabbit, boar) or the Kruskal–Wallis test with Bonferroni correction (bovine), as determined by normality assumptions (Shapiro–Wilk test). Significant differences at *p* < 0.01 (**) in bovine samples between ejaculated vs. selected and selected vs. acrosome-reacted.

DIA-NN filtered reports were used for quantifying ZAN, in which abundance values represented median peak areas. ZAN was observed in every experimental condition for each species (Figure 5). While no significant differences were detected across conditions in rabbit and boar samples (one-way ANOVA, *p* > 0.05), bovine sperm showed significant variations (Kruskal–Wallis, *p* < 0.05). Specifically, rabbit samples exhibited an area of 360,601.00 in ejaculated sperm, 285,123.92 in selected sperm via swim-up in capacitation medium and 206,755.46 in acrosome-reacted sperm. Turning to boar samples, proteomic analysis revealed an area of 121,939.47 in ejaculated sperm. Following swim-up selection in capacitation medium, this value increased to 197,038.60, whereas induction of acrosome reaction resulted in a decrease to 154,760.00. Although no significant differences were observed among experimental conditions in boar sperm, the data showed a nonsignificant trend (one-way ANOVA, *p* = 0.065). A similar trend was observed in bull sperm samples, where the area increased significantly from 253,020.00 in ejaculated sperm to 287,136.00 after selection (Bonferroni-corrected; *p* < 0.01) and declined significantly to 236,566.00 following acrosome reaction induction (Bonferroni-corrected; *p* < 0.01).

## 3. Discussion

During capacitation, a subset of proteins is translocated from the acrosomal membrane to the anterior region of the plasma membrane, where these proteins may contribute to zona pellucida recognition. According to Tanphaichitr (2015) [19], ZAN is one of the most abundant proteins within these protein complexes. Although previous studies have supported its involvement in zona pellucida recognition, its presence after the acrosome reaction has remained largely unexplored. Therefore, we investigated whether ZAN is redistributed following the acrosome reaction. To provide a comparative framework, we examined two species belonging to Laurasiatheria (boar and bull) and one belonging to Euarchontoglires (rabbit), all within Boreutheria.

Consistent with previous reports, ZAN was detected in the anterior region of intact spermatozoa in all three species studied. However, the use of an antibody against a region overlapping the MAM region, rather than the regions known as ‘active’ D0–D3, allowed us to detect ZAN localization in the post-acrosomal region of both intact and reacted spermatozoa. Analysis of intact spermatozoa (the majority group subjected to E and S conditions) revealed distinct patterns of ZAN localization based on the species studied. In rabbits and bulls, ZAN was detected in the acrosomal region (apical ridge + pre-equatorial subdomain), either with or without the post-acrosomal region (patterns 1 and 2, respectively). Meanwhile, intact boar spermatozoa showed ZAN in the pre-equatorial and post-acrosomal domains, with or without intense labeling along the apical ridge. This specific labeling of the apical region is consistent with the model proposed by Tanphaichitr et al. [19], which describes the relocation of proteins involved in ZP interactions in porcine sperm. Moreover, this protein dynamic has also been observed in boars by our group with the IZUMO1 protein [18], which is involved in both adhesion and fusion of gametes.

With regard to the reacted spermatozoa, we observed that despite variable ZAN loss according to the species, it is largely retained in the post-acrosomal region (Figure 4). It is important to note that ZAN is found in some rabbit sperm in a symmetrical thickening above the equatorial segment (equatorial bulges, pattern 3) [20]. This morphological specialization of the equatorial segment could increase the available contact surface, potentially facilitating adhesion and fusion between sperm–oocyte membranes. In addition, it may help maintain proper orientation of the sperm when it contacts the oolemma, promoting efficient fusion.

Complementarily, an exploratory assay performed on porcine spermatozoa ( Video S1) revealed an asymmetric localization of ZAN, restricted to one side of the post-acrosomal region. This polarized distribution is particularly noteworthy considering the characteristic tangential interaction between mammalian spermatozoa and the oocyte. Rather than contacting the oolemma perpendicularly, mammalian spermatozoa are thought to maintain a defined dorsoventral orientation and establish membrane contact at an oblique angle [21]. The asymmetric localization of ZAN is therefore consistent with the existence of a dorsoventrally polarized sperm head and raises the possibility that ZAN is associated with a specialized membrane domain that engages the oolemma interaction during the final stages of fertilization.

Quantitative proteomic profiling by LC–MS/MS revealed species-specific changes in ZAN abundance during the acquisition of fertilization competence (Figure 5). Proteomics studies have demonstrated that sperm capacitation is accompanied by extensive changes in the abundance, modification, and membrane association of sperm proteins, reflecting the substantial molecular remodeling that occurs during this process [22,23,24]. In our study, bull sperm exhibited a statistically significant increase in ZAN abundance following the swim-up selection in capacitation medium, whereas boar samples showed a slight, nonsignificant increase consistent with effective selection. In rabbit sperm, swim-up selection in capacitation medium was associated with a reduction in population variability rather than an increase in ZAN abundance. These interspecific differences may reflect species-specific regulation of sperm protein composition during the acquisition of fertilization competence, a process that in vivo involves sperm selection and capacitation within the female reproductive tract.

Following induction of acrosome reaction, ZAN abundance decreased consistently across all species studied, although the magnitude and statistical significance of this decrease varied among them. This quantitative reduction aligns with our fluorescence data (Figure 4), suggesting that the reduction in detectable ZAN may reflect protein redistribution or release during acrosomal exocytosis. Such changes are compatible with the extensive remodeling of sperm surface and acrosomal proteins that accompanies the acrosome reaction [25,26,27].

**Figure 6:**
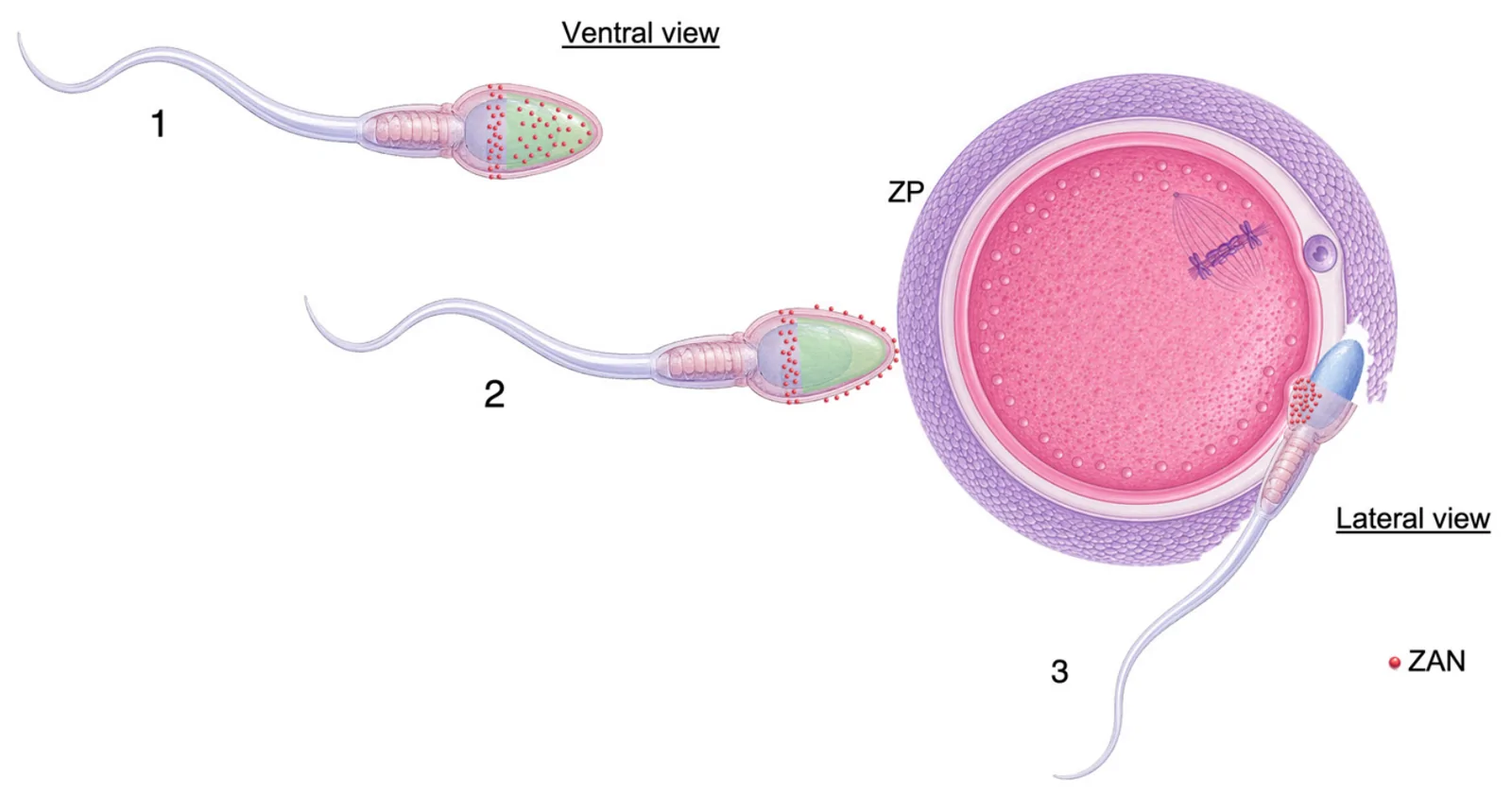
Proposed model of ZAN localization during mammalian fertilization in rabbit, boar, and bull. (1) In ejaculated spermatozoa, ZAN is predominantly localized in the pre-equatorial region, and may also be detected in the post-acrosomal region depending on the species. (2) Following swim-up selection in capacitation medium, ZAN becomes exposed at the sperm surface, allowing its interaction with the zona pellucida (ZP). (3) After acrosomal exosis, ZAN remains on the ventral side of the post-acrosomal region, where it may participate in the interaction between the sperm plasma membrane and the oolemma.

Taken together, the fluorescence analyses, three-dimensional reconstruction, and proteomic data provide complementary evidence supporting a model of ZAN localization during fertilization (Figure 6). This model illustrates, for the first time, the conserved retention of ZAN after the acrosome reaction and provides a conceptual framework linking its spatial distribution to its proposed role during the final stages of fertilization across rabbit, boar and bull. Further studies are needed to determine the functional significance of this post-acrosomal localization and to establish whether ZAN contributes directly to subsequent sperm–oocyte interactions.

## 4. Materials and Methods

### 4.1. Experimental Design

**Figure 7:**
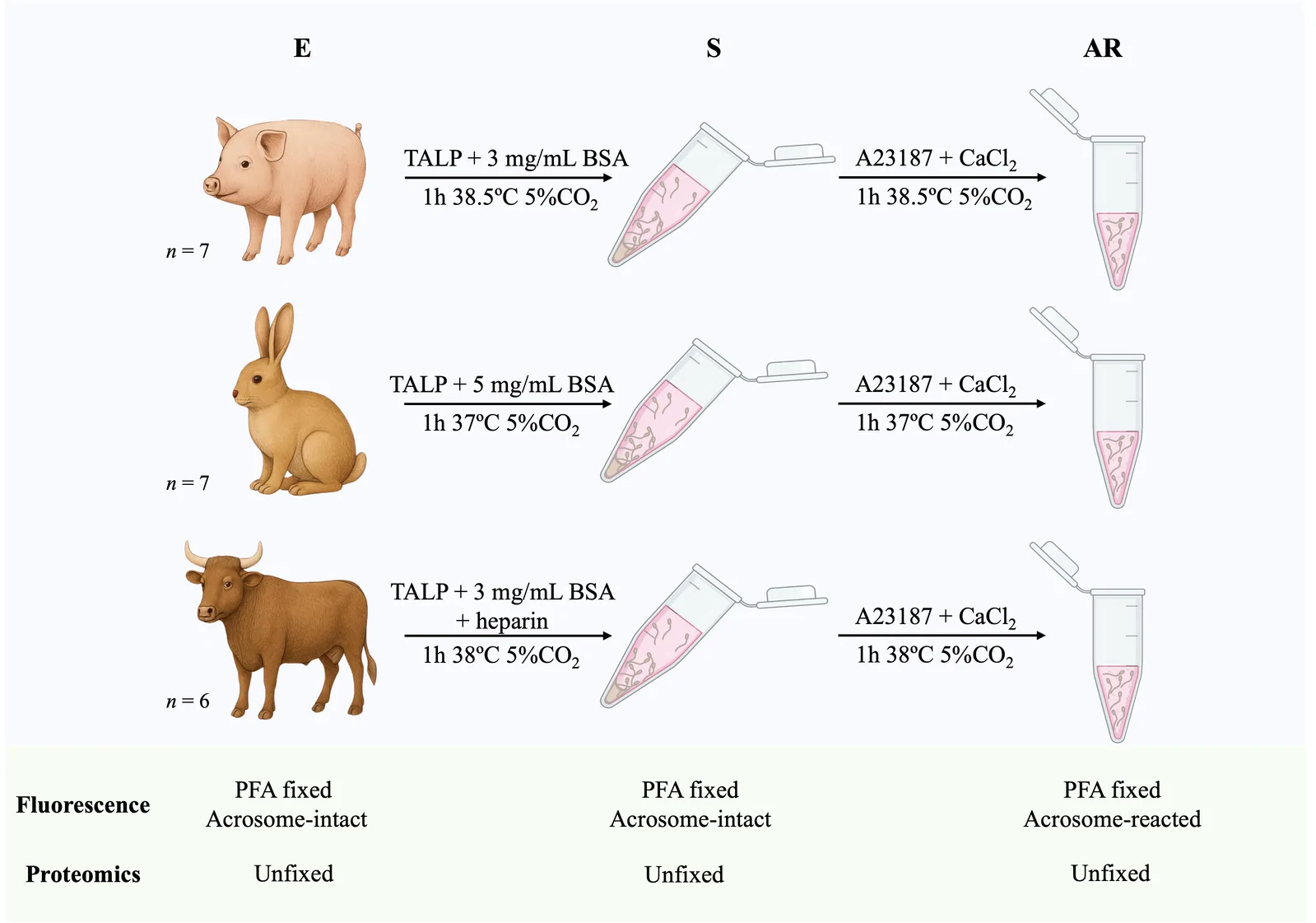
Schematic description of the experimental design followed. Ejaculated spermatozoa (E); spermatozoa selected by swim-up in capacitation medium (S); spermatozoa subjected to acrosome reaction induction (AR); paraformaldehyde (PFA).

Ejaculates from three different species (boar, rabbit, and bull) were processed under the following conditions (Figure 7): ejaculated spermatozoa (E), spermatozoa selected by swim-up in capacitation medium (S), and spermatozoa subjected to acrosome reaction induction (AR). In all conditions, double labeling was performed to determine ZAN distribution and to classify the acrosomal status using PNA-FITC (acrosome-intact vs. acrosome-reacted). In the pre- and post-selection conditions (E and S), spermatozoa with intact acrosomes and spontaneous-reacted acrosomes were evaluated, whereas in the acrosome reaction-induced condition, only acrosome-reacted spermatozoa were evaluated. Additionally, unfixed aliquots from each of the three conditions of each species were subjected to quantitative proteomic analysis by LC-MS/MS.

### 4.2. Sample Preparation

Boar semen (*n* = 7; Pietrain, Axiom Genetics line; purchased from SPERMATICA REPRODUCCION SL., Lorca, Spain) was collected using the gloved hand technique and packaged in bags concentrated at 30 × 10^6^ spermatozoa/mL in Zoosperm ND10 (TECNOVET SL., Barcelona, Spain). Rabbit semen (*n* = 7; New Zealand White) was kindly provided by the coordinating project (PID2021/123091NB/C-21) and was collected using an artificial vagina (IMV Technologies, L’Aigle, France) in the animal facility of the University of Murcia under the approval of the Ethics Committee of the University of Murcia (protocol code CEEA 775/2021). Commercial cryopreserved bull semen (*n* = 6; Limousin; Aberekin S.A., Bizkaia, Spain) was thawed by immersion in a 37 °C water bath for 30 s. Before sample processing, sperm quality was assessed by evaluating concentration and motility using the AI Station computer-assisted sperm analysis (CASA) system (version 1.3.57, Sperm Analysis Technologies S.L., Spermtech, Valencia, Spain), viability using VitalStain^TM^ (NidaCon International AB, Mölndal, Sweden), and morphology using SpermBlue (Microptic, Barcelona, Spain). Semen from each species was centrifuged and resuspended in non-capacitation medium at 10 × 10^6^ spermatozoa/mL. The inclusion criteria for ejaculate selection were: total motility > 70%, viable sperm > 90%, and morpho-anomalies ≤ 30%.

### 4.3. Sperm Selection via Swim-Up Capacitation Medium

The swim-up procedure [28] consisted of placing 100 μL of the semen sample at the bottom of an Eppendorf tube containing 1 mL of capacitation medium (see Table A1). The sample was then incubated at the appropriate temperature (boar 38.5 °C, rabbit 37 °C and bull 38 °C) with 5% (*v*/*v*) CO_2_ and at a 45° angle for 1 h. Finally, the supernatant fraction (900 μL) was collected and washed three times with sterile-filtered Dulbecco’s phosphate-buffered saline without calcium, magnesium, and phenol red (PBS, Capricorn Scientific GmbH, Ebsdorfergrund, Germany) by centrifugation (200× *g*, 5 min).

### 4.4. Induction and Evaluation of Acrosomal Status

The acrosome reaction was induced by adding 10 μM of calcium ionophore A23187 (Sigma-Aldrich^®^, Saint Louis, MO, USA) and 2 mM of calcium chloride (Panreac Química S.L.U, Barcelona, Spain) at the appropriate temperature (boar 38.5 °C, rabbit 37 °C and bull 38 °C) with 5% (*v*/*v*) CO_2_ for 1 h, following previous protocols from our group [29,30].

The samples were fixed on coverslips with methanol for 30 min. Then, the cells were washed three times with phosphate-buffered saline (PBS) and incubated with *Peanut agglutinin* lectin conjugated with fluorescein-5-isothiocyanate (PNA-FITC, Vector Laboratories, Burlingame, CA, USA) at a final concentration of 5 μg/mL for 30 min. After three washes, the samples were mounted using Fluoroshield^TM^ with 4′,6-diamidine-2′-phenylindole dihydrochloride (Sigma-Aldrich^®^, Saint Louis, MO, USA).

### 4.5. Fixation

All sperm physiological conditions (E, S, and AR) were divided into two aliquots. One of them was fixed in 2% (*w*/*v*) paraformaldehyde (Electron MicroscopySciences, Hatfield, PA, USA) diluted in PBS for 45 min at 4 °C. Afterward, the fixative solution was replaced with PBS to reach a final concentration of 10 × 10^6^ spermatozoa/mL, and the samples were stored at 4 °C until use. The other aliquot was stored unfixed at −80 °C for proteomic analysis.

### 4.6. ZAN Immunostaining

A total of 5 μL of each paraformaldehyde-fixed sample were placed on a coverslip. When the smear was dry, cells were washed twice in PBS for 5 min. Then, the smears were permeabilized with 0.2% (*w*/*v*) Triton X-100 for 10 min and blocked for 15 min in 0.2% (*w*/*v*) BSA-PBS. Afterward, smears were incubated overnight at 4 °C with a rabbit polyclonal anti-ZAN antibody (1:100, orb158733, Biorbyt Ltd., Cambridge, UK), raised against a KLH-conjugated synthetic peptide corresponding to amino acids 201–300 of human ZAN. To assess cross-species conservation of the immunogenic region, the sequences were compared using BLASTp via the NCBI web interface (https://blast.ncbi.nlm.nih.gov/Blast.cgi?PROGRAM=blastp; accessed on 5 August 2025).

Subsequently, smears were washed and incubated with a secondary anti-rabbit antibody conjugated with Cy^3^ (1:100, AB_2338000, Jackson ImmunoResearch, Ely, UK) for 1 h at room temperature (RT) in the dark. For the co-staining of ZAN and acrosome status, samples were first incubated overnight with anti-ZAN followed by 30 min incubation with PNA-FITC. Finally, the smears were washed and mounted using Fluoroshield^TM^ with DAPI. Negative controls were performed by omitting the primary antibody.

At least 200 cells were evaluated under each condition using a Confocal Laser Scanning Zeiss LSM 800 Microscope (Zeiss, Oberkochen, Germany) with an oil 100× objective. Images were acquired using 405, 488, and 561 nm laser lines, with laser powers of 1.3%, 0.30%, and 0.30%, respectively. The pinhole was set to 43 µm and the master gain to 650 V. The same acquisition settings were maintained across all species and experimental conditions. The ZAN staining patterns in the sperm head and acrosome were quantified as percentages (%).

### 4.7. LC MS/MS Analysis

The proteomic analysis was performed in the proteomics facility of SCSIE University of Valencia. This proteomics laboratory is a member of Proteored. In-solution samples of six randomly selected males from each species (rabbit, boar, and bull) under all experimental conditions (ejaculated spermatozoa, spermatozoa selected by swim-up in capacitation medium, and spermatozoa subjected to acrosome reaction induction) were used. The samples from rabbit and bull were processed following the LC-MS/MS workflow previously established for boar samples [18]. All analytical parameters and quality control criteria were kept consistent with the original protocol to ensure methodological reproducibility across species.

### 4.8. Statistical Analysis

Statistical analyses were conducted using IBM SPSS Statistics 28.0 (IBM, Armonk, Westchester County, NY, USA). The Shapiro–Wilk test showed that ZAN location was normally distributed for each sperm physiological condition (E and S) in boar and bull, whereas it was not normally distributed in rabbit. Homogeneity of variances was evaluated using Levene’s test when parametric analyses were applicable. Variables following a normal distribution were analyzed by two-tailed Student’s t-test. In cases where the assumption of homogeneity of variance was violated, the Welch correction was applied. To ensure the robustness of the parametric findings, all comparisons conducted using t-tests were additionally verified using the nonparametric Mann–Whitney U test, yielding the same overall results. Variables that did not follow a normal distribution were analyzed by nonparametric Mann–Whitney’s U tests. Statistical significance was set at *p* < 0.05 for all analyses. In the analysis of the area data obtained with DIA-NN, the rabbit and the boar datasets showed normal distributions, whereas the bull dataset did not. One-way ANOVA was used for species with normal distributions, whereas the Kruskal–Wallis test with post hoc Bonferroni correction was used for species with nonnormal distributions.

## 5. Conclusions

Collectively, this multispecies approach provides evidence that ZAN retention following the acrosome reaction is conserved across the mammalian species examined. Rather than being completely lost during acrosomal exocytosis, ZAN remains associated with membrane domains involved in the final stages of fertilization. Its persistence within the equatorial segment or post-acrosomal region, together with the asymmetric localization observed in porcine spermatozoa, is consistent with the functional polarization of the sperm head and suggests that ZAN may contribute to the directional interaction between the sperm plasma membrane and the oolemma during gamete binding and/or fusion.

## Data Availability

The data supporting the findings of this study are contained within the article.

## Supplementary Materials

The following supporting information can be downloaded at: https://www.mdpi.com/article/10.3390/ijms27188062/s1.

## Appendix A

**Table A1:** TALP medium composition.

Reagent	Rabbit	Boar	Bull
NaCl	114.06 mM	114.06 mM	114.06 mM
KCl	3.20 mM	3.20 mM	3.20 mM
NaH_2_PO_4_	0.35 mM	0.35 mM	0.35 mM
CaCl_2_	-	-	2.10 mM
MgCl_2_·6H_2_O	0.50 mM	0.5 mM	0.50 mM
Glucose	5.00 mM	5.00 mM	-
NaHCO_3_	25.07 mM	25.07 mM	-
Caffeine	2 mM	2.00 mM	-
Calcium lactate pentahydrate (C_6_H_10_CaO_6_·5H_2_O)	8.00 mM	8.00 mM	-
Na pyruvate	1.10 mM	1.10 mM	1.10 mM
Sodium lactate (NaC_3_H_5_O_3_)	18.00 mM	18 mM	18.00 mM
PVA	1.00 mg mL^−1^	1.00 mg mL^−1^	-
Kanamycin sulphate	0.17 mM	0.17 mM	0.17 mM
Phenol red	0.003 mM	0.003 mM	0.003 mM
BSA	5.00 mg mL^−1^	3.00 mg mL^−1^	3.00 mg mL^−1^
Heparin			0.01 mg mL^−1^

Rabbit and boar TALP media were previously pre-equilibrated overnight at the appropriate temperature, 5% (*v*/*v*) CO_2_, 95% humidified air and with a final pH of 7.4. Bovine TALP medium was pre-warmed to the appropriate temperature before use. All those reagents were purchased from Sigma (Sigma-Aldrich^®^, Saint Louis, MO, USA) and were embryo and/or cell culture tested.

## References

[1] Hardy D.M., Garbers D.L. (1995). A Sperm Membrane Protein That Binds in a Species-Specific Manner to the Egg Extracellular Matrix Is Homologous to von Willebrand Factor. J. Biol. Chem..

[2] Herlyn H., Zischler H. (2008). The Molecular Evolution of Sperm Zonadhesin. Int. J. Dev. Biol..

[3] Hickox J.R., Bi M., Hardy D.M. (2001). Heterogeneous Processing and Zona Pellucida Binding Activity of Pig Zonadhesin. J. Biol. Chem..

[4] Bi M., Hickox J.R., Winfrey V.P., Olson G.E., Hardy D.M. (2003). Processing, Localization and Binding Activity of Zonadhesin Suggest a Function in Sperm Adhesion to the Zona Pellucida during Exocytosis of the Acrosome. Biochem. J..

[5] Gao Z., Garbers D.L. (1998). Species Diversity in the Structure of Zonadhesin, a Sperm-Specific Membrane Protein Containing Multiple Cell Adhesion Molecule-like Domains. J. Biol. Chem..

[6] Tardif S., Wilson M.D., Wagner R., Hunt P., Gertsenstein M., Nagy A., Lobe C., Koop B.F., Hardy D.M. (2010). Zonadhesin Is Essential for Species Specificity of Sperm Adhesion to the Egg Zona Pellucida. J. Biol. Chem..

[7] Gao Z., Harumi T., Garbers D.L. (1997). Chromosome Localization of the Mouse Zonadhesin Gene and the Human Zonadhesin Gene (ZAN). Genomics.

[8] Lea I.A., Sivashanmugam P., O’Rand M.G. (2001). Zonadhesin: Characterization, Localization, and Zona Pellucida Binding1. Biol. Reprod..

[9] Olson G.E., Winfrey V.P., Bi M., Hardy D.M., NagDas S.K. (2004). Zonadhesin Assembly into the Hamster Sperm Acrosomal Matrix Occurs by Distinct Targeting Strategies during Spermiogenesis and Maturation in the Epididymis. Biol. Reprod..

[10] Tardif S., Cormier N. (2011). Role of Zonadhesin during Sperm-Egg Interaction: A Species-Specific Acrosomal Molecule with Multiple Functions. Mol. Hum. Reprod..

[11] Cohen D.J., Maldera J.A., Vasen G., Ernesto J.I., Muñoz M.W., Battistone M.A., Cuasnicú P.S. (2011). Epididymal Protein CRISP1 Plays Different Roles during the Fertilization Process. J. Androl..

[12] Teves M.E., Roldan E.R.S. (2022). Spermbauplan and Function and Underlying Processes of Sperm Formation and Selection. Physiol. Rev..

[13] Stival C., Puga Molina L.d.C., Paudel B., Buffone M.G., Visconti P.E., Krapf D., Buffone M.G. (2016). Sperm Capacitation and Acrosome Reaction in Mammalian Sperm. Sperm Acrosome Biogenesis and Function During Fertilization.

[14] Darszon A., Nishigaki T., López-González I., Visconti P.E., Treviño C.L. (2020). Differences and Similarities: The Richness of Comparative Sperm Physiology. Physiology.

[15] Rothschild M.F. (1996). Genetics and Reproduction in the Pig. Anim. Reprod. Sci..

[16] Thibier M. (2005). The Zootechnical Applications of Biotechnology in Animal Reproduction: Current Methods and Perspectives. Reprod. Nutr. Dev..

[17] Gidenne T., Garreau H., Drouilhet L., Aubert C., Maertens L. (2017). Improving Feed Efficiency in Rabbit Production, a Review on Nutritional, Technico-Economical, Genetic and Environmental Aspects. Anim. Feed. Sci. Technol..

[18] Hernández-Falcó M., Sáez-Espinosa P., López-Botella A., Robles-Gómez L., García-Vázquez F.A., Izquierdo-Rico M.J., Llamas-López P.J., Gómez-Torres M.J. (2025). Immunolocalization and Proteomic Analyses of IZUMO1 in Porcine Spermatozoa. Front. Cell Dev. Biol..

[19] Tanphaichitr N., Kongmanas K., Kruevaisayawan H., Saewu A., Sugeng C., Fernandes J., Souda P., Angel J.B., Faull K.F., Aitken R.J. (2015). Remodeling of the Plasma Membrane in Preparation for Sperm-Egg Recognition: Roles of Acrosomal Proteins. Asian J. Androl..

[20] Bernstein M.H., Teichman R.J. (1972). Regional Differentiation in the Heads of Spermatozoa of Rabbit, Man and Bull. Am. J. Anat..

[21] Bedford J.M. (2006). Why Do Penetrating Sperm Create an Oblique Path in the Zona Pellucida. Reproduction.

[22] Sleight S.B., Miranda P.V., Plaskett N.-W., Maier B., Lysiak J., Scrable H., Herr J.C., Visconti P.E. (2005). Isolation and Proteomic Analysis of Mouse Sperm Detergent-Resistant Membrane Fractions: Evidence for Dissociation of Lipid Rafts During Capacitation1. Biol. Reprod..

[23] Castillo J., Bogle O.A., Jodar M., Torabi F., Delgado-Dueñas D., Estanyol J.M., Ballescà J.L., Miller D., Oliva R. (2019). Proteomic Changes in Human Sperm During Sequential in vitro Capacitation and Acrosome Reaction. Front. Cell Dev. Biol..

[24] Baker M.A. (2016). Proteomics of Post-Translational Modifications of Mammalian Spermatozoa. Cell Tissue Res..

[25] Cross N.L. (2004). Reorganization of Lipid Rafts during Capacitation of Human Sperm. Biol. Reprod..

[26] Gadella B.M., Tsai P.-S., Boerke A., Brewis I.A. (2008). Sperm Head Membrane Reorganisation during Capacitation. Int. J. Dev. Biol..

[27] Sosa C.M., Pavarotti M.A., Zanetti M.N., Zoppino F.C.M., De Blas G.A., Mayorga L.S. (2015). Kinetics of Human Sperm Acrosomal Exocytosis. Mol. Hum. Reprod..

[28] Boiti C., Castellini C., Theau-Clement M., Besenfelder U., Liguori L., Renieri T., Pizzi F. (2005). Guidelines for the Handing of Rabbit Bucks and Semen. World Rabbit Sci..

[29] Robles-Gómez L., González-Brusi L., Sáez-Espinosa P., Huerta-Retamal N., Cots-Rodríguez P., Avilés M., Gómez-Torres M.J. (2021). Specific Lectin Binding Sites during in vitro Capacitation and Acrosome Reaction in Boar Spermatozoa. Ital. J. Anim. Sci..

[30] Sáez-Espinosa P., Torrijo-Boix S., Huerta-Retamal N., Avilés M., Aizpurua J., Romero A., Gómez-Torres M.J. (2018). La Capacitación y La Reacción Acrosómica Se Asocian Con Cambios En La Localización Del Ácido Siálico y En La Morfometría de La Región Cefálica Del Espermatozoide Humano. Rev. Int. Androl..

